# Quality of Mother-Child Interaction Before, During, and After Smartphone Use

**DOI:** 10.3389/fpsyg.2021.616656

**Published:** 2021-03-29

**Authors:** Carolin Konrad, Mona Hillmann, Janine Rispler, Luisa Niehaus, Lina Neuhoff, Rachel Barr

**Affiliations:** ^1^Clinical Child and Adolescent Psychology, Faculty of Psychology, Mental Health Research and Treatment Center, Ruhr University Bochum, Bochum, Germany; ^2^Department of Psychology, Georgetown University, Washington, DC, United States

**Keywords:** technoference, parent-child interaction, still face, interactional quality, interruption, smartphone, media use

## Abstract

Studies have demonstrated that parents often exhibit a still face while silently reading their cell phones when responding to texts. Such disruptions to parent-child interactions have been observed during parental media use such as texting and these disruptions have been termed technoference. In the present study, we explored changes to mother-child interactions that occur before, during and after interruptions due to texting using an adapted naturalistic still face paradigm. Specifically, we examined the effect of an interruption due to either maternal smartphone use or use of an analog medium on maternal interaction quality with their 20- to 22-month-old children. Mother-child interactions during free play were interrupted for 2 min by asking the mothers to fill out a questionnaire either (a) by typing on the smartphone (smartphone group) or (b) on paper with a pen (paper-pencil group). Interactional quality was compared between free-play and interruption phases and to a no-interruption control group. Mixed ANOVA across phase and condition indicated that maternal responsiveness and pedagogical behavior decreased during the interruption phase for both the interruption groups (smartphone and paper-and-pencil) but not for the no-interruption group. Children also increased their positive bids for attention during the paper-and-pencil and the smartphone conditions relative to the no-interruption control. These findings are consistent with a large body of research on the still-face paradigm and with a recent study demonstrating that smartphone interruptions decreased parenting quality. The present study, however, connects these lines of research showing the many everyday disruptions to parent-child interactions are likely to decrease parenting quality and that toddlers are likely to detect and attempt to repair such interruptions.

## Introduction

The quality of early mother-child interactions during play contributes to both child development and the mother-child relationship (e.g., Tamis-LeMonda et al., 2001; Landry et al., 2006; Ginsburg, 2007). Playing together offers mothers and their toddlers a unique opportunity for language-rich interactions and emotional engagement (Ginsburg, 2007; Yogman et al., 2018). Furthermore, there is evidence that the quality of such early mother-child interactions—especially maternal responsiveness as a prompt, contingent, and appropriate reaction to child behavior (Bornstein et al., 2008)—is a powerful predictor of social-emotional, cognitive, and linguistic child development and the parent-child bond (Ainsworth and Bell, 1974; Tamis-LeMonda et al., 2001; Landry et al., 2006).

It is evident that during the digital age where mobile technology is ubiquitous that such interactions are shaped by digital media (Radesky J. S. et al., 2015; Barr and Linebarger, 2017; Rideout, 2017; McDaniel, 2019; Vanden Abeele et al., 2020; Wolfers et al., 2020). New mobile devices, including smartphones, differ from traditional digital media (e.g., TVs) in that parents and their children can take them with them wherever they go and use them in a variety of ways at any time (Wartella, 2019). Thus, mobile media enable parents to spend time with their children and at the same time be available for friends or professional partners (Radesky et al., 2016a; Mangan et al., 2018). As a result, parents use smartphones a significant proportion of the time in everyday family situations, in the presence of their small children, for example during play, meal, and bedtime routines (McDaniel and Coyne, 2016; Yuan et al., 2019; Barr et al., 2020; Vanden Abeele et al., 2020; Wolfers et al., 2020). With the ubiquity of smartphones in everyday family life, there is a risk that mother-infant interactions will be interrupted and qualitatively impaired. Smartphone use during interactions results in repeated disconnections between social partners which has recently been labeled “technoference” (McDaniel and Radesky, 2018). Infants might be especially sensitive to those disruptions because they resemble a classical still face (Myruski et al., 2018). As a result, mothers of toddlers report experiencing smartphone interruptions during interactions with their toddlers—which they report are either self-initiated or due to device notifications (McDaniel and Coyne, 2016; Newsham et al., 2020).

Frequent smartphone checking and pickups interrupts early mother-child interactions and impairs the quality, because mothers may be less responsive. Such repeated interruptions to play could have negative consequences for the mother-child relationship. It is therefore particularly important to examine the effect of maternal smartphone use on the quality of mother-child interactions. Initial, predominantly qualitative observational studies provide evidence that parental smartphone use affects parent-child interactions. Observational studies conducted in a fast food restaurant (Radesky et al., 2014) and on playgrounds (Hiniker et al., 2015; Abels et al., 2018; Lemish et al., 2019; Vanden Abeele et al., 2020; Wolfers et al., 2020) found that parents who were immersed in smartphone use communicated less frequently with their children and were less responsive to children's needs and attention-seeking behavior. Sometimes the parents appeared to be annoyed (Lemish et al., 2019) or even hostile after repeated child attempts to call parental attention (Radesky et al., 2014). Rather than just the frequency of use, the duration of use was also a factor. Wolfers et al. (2020) reported that mothers who were observed on play-grounds using their smartphones for longer were also rated lower on maternal sensitivity. A laboratory study simulating a mealtime situation found that mothers who used their mobile device spontaneously during a structured situation were less likely to initiate verbal and non-verbal interactions with their children (Radesky J. et al., 2015). Interestingly, it was observed that other parental activities on playgrounds (reading a magazine, talking to another person) also absorbed parents and impaired parental responsiveness, but not as much as parental smartphone use (Abels et al., 2018; Lemish et al., 2019; Vanden Abeele et al., 2020). Taken together, these findings suggest that immersive smartphone use by parents disrupt every-day routine parent-child interactions.

There have been few experimental studies, however, that have investigated how smartphone use might disrupt interactions. In an experimental study, Reed et al. (2017) tested whether cell phone calls interrupted language learning by 2-year-olds. Using a within-subjects design, 38 mothers taught their 2-year-olds two novel words. Mothers received a call that interrupted them while teaching one of the words, but for the other word the call occurred prior to teaching. Children were significantly more likely to learn the uninterrupted word than the interrupted word. This finding remained despite the fact that the mother taught the word the same number of times on average in both conditions. However, this was a phone call interruption and it is not clear whether a text that simply involves silent reading and responding would be as disruptive.

When checking mobile phones, parents' faces frequently have no expression, and these periods of time may be perceived by young children as a “still face,” to which children respond aversively (Adamson and Frick, 2003). The “still face” presented to the infant during smartphone use may disrupt communication from the infant to the parent as well. Using a standard still-face paradigm, Goldstein et al. (2009) reported that parents respond to infant vocalizations 30 to 50% of the time. When parents present a “still face” response, 5-month-olds increase their vocalizations, presumably to regain the adult's attention. When the interaction resumes, the infant decreases vocalizations and re-engages in turn-taking with the parent. The greater the increase in vocalizations in response to a “still face' the better language outcomes are at 1 year of age.

In an experimental study, while parents were teaching their infants how to make a rattle, parents were interrupted by a text asking them to complete a questionnaire instead. In this study, parents were randomly assigned to one of four experimental conditions: interruption-first condition, one-interruption condition (occurred in the middle), three-interruptions condition and a no-interruption condition (Konrad et al., 2021). Parents demonstrated how to make the rattle 4 times. Text interruptions occurred before or between demonstrations. After the demonstration was complete, infants were given the opportunity to make the rattle themselves. Their performance was compared to a baseline control condition who had not seen how to make the rattle. Most parents (77%) exhibited a still face during the text interruption. Despite this brief period of technoference, infants in all experimental groups performed significantly above the baseline control condition, showing evidence of learning from the parental demonstration.

Finally, researchers used a modified version of the still face procedure to examine changes in interactional quality during smartphone use. Myruski et al. (2018) instructed 50 mothers of 7- to 23-month-olds to assume a still face when looking at a smartphone during a 2 min interruption to a free play period. In the first phase of the study, mothers played freely with their infants for 5 min. Then in the still face phase, they were asked to interact only with their phone for 2 min. Finally, there was a one minute second free play phase, called the reunion phase. The authors reported that there were changes in infant exploration which was highest during phase 1 free play and decreased during the still face and reunion phases of the study. During the adapted mobile device still face phase, infants exhibited the typical protest and distress response exhibited in other versions of the still-face paradigm (Myruski et al., 2018). Myruski et al. (2018), asked parents 4 self-report questions to assess habitual mobile device use. More frequent habitual maternal mobile device use was correlated with less engagement with the mother during reunion. However, there were a number of limitations to the study. The reunion phase was shorter than the initial free play phase, making it difficult to compare between the initial free play and the reunion phases. Although mothers were instructed to interact with their phones and not with their infants they did not receive a text and were not reading or responding to a text. There were no other control conditions. An open question is whether other non-media related absorbing activities that limit maternal responsiveness trigger negative child emotions comparable to that of maternal mobile device use.

In summary, these studies provide initial indications that parental smartphone use has a particularly negative impact on parental interaction quality and attention to their children compared to other activities that engage them. Researchers have not previously investigated whether smartphone use can lead to changes in maternal behavior. Furthermore, they have not tested the assumption that smartphone use is more disruptive for an interaction than other non-digital media. Here, we explored changes to mother-child interactions that occur before, during and after interruptions due to texting. We wanted to examine how parents would typically respond to their infants while answering texts on a smartphone. We did not specifically try to replicate a still face paradigm. We did not explicitly instruct them to assume a still face but rather used a detailed coding scheme to code parent behavior before, during, and after the interruption. Following previous studies we also coded child behavior. We compared the effect of maternal smartphone use to an analog medium on maternal interaction quality with their 20- to 22-month-old children. Following the Myruski et al. (2018) procedure, there was a free play period followed by a 2-min interruption. During the interruption, mothers were asked to fill out a questionnaire either a) by typing on the smartphone (smartphone group) or b) on paper with a pen (paper-pencil group). The interruption groups were compared to a no-interruption group.

We hypothesized an interaction effect between phase and condition. For the interruption conditions we hypothesized that there would be a u-shaped function in interactions with high rates of positive mother-child interactions and child behavior during the first free play period, a decrease in positive interactions during the interruption phase and an increase in positive interaction in phase 3. We hypothesized that the interaction quality and the child's behavior would remain constant in the no-interruption group across phases. Focusing on the interruption phase, we hypothesized that the quality of interaction in the no-interruption group would be higher than in the paper-pencil group and higher in the paper-pencil group than in the smartphone group. Likewise, we hypothesized that child negative affect, social bids and forbidden behavior would increase during the interruption phase, but more so in the smartphone than in the paper-pencil condition and least in the no-interruption group.

## Methods

### Participants

We conducted an a priori power analysis for a mixed ANOVA for an effect size of 0.25 (Myruski et al., 2018), power of 0.95, alpha 0.05, 3 groups and 3 measurements and received a required sample size of 54. Fifty-four full-term, healthy 20-22-month-old infants (*M*_*age*_ = 20.8 months, *SD* = 0.5 months) were randomly assigned to one of two interruption conditions (smartphone: *n* = 18, 10 female; paper-pencil: *n* = 20, 9 female) or a no-interruption control condition (*n* = 16, 8 female). Three additional families participated, but had to be excluded from analysis due to technical difficulties (*n* = 1) or the interruption being too long (*n* = 2).

Mothers were on average 33.8 years (*SD* = 3.6 years, range = 24–42 years), well-educated (62% had a university degree as the highest educational qualification), and German nationals (88.7% were of German nationality, 3.8% had a nationality different from German, and 7.5% had a German and another nationality, 53 mothers reporting). Mothers also reported family's yearly income before taxes (44 reporting). 2.3% had <10.000€, 18.2% had 20.000€ to 39.000€, 25% had 40.000–59.000€, 27.2% had 60.000–80.000€, 15.9% had more than 80.000€, 11.4% preferred not to answer the question. 100% of the mothers owned a smartphone (51 mothers reporting). The families were recruited via the local birth register of the city of Bochum. The children received a small book and a certificate and the mothers received 5€. The study was approved on January 27, 2019 by the local ethics committee of the Faculty of Psychology at the Ruhr University Bochum. Data was collected between February and August, 2019.

### Design

Mothers were instructed to play with a standard set of toys. Analogous to the standard phases of a still face procedure, there were three phases of the study for the smart-phone and paper-and-pencil interruption conditions. There was an initial 3 min free play period, followed by a 2 min interruption phase and a second 3 min free play period. The no-interruption group had an 8 min uninterrupted free play period.

### Material

#### CAFE Media Assessment Questionnaire

Parents completed a 74-item Qualtrics survey covering 10 topics, including household composition and demographics, parental mediation of media use, parent attitudes toward media use, and access to and regularity of use of different devices frequently used in the modern household (Barr et al., 2020) (average time to complete in our sample ~30 min). The questions were derived from a number of existing surveys (e.g., Lapierre et al., 2012; Rideout, 2017) and were updated to reflect current technologies and research on the content and context of early media exposure. Established measures of parent media use, behaviors, and attitudes, such as Valkenburg et al. (1999) parent mediation scale are also part of the survey. Since we were especially interested in maternal smartphone use in this study, we selected the following items for the analyses: duration of phone use on typical weekdays (scale from 1 to 8: “1” = never, “2” = <30 min, “3” = 30-60 min, “4” = 1-2 h, “5” = 2-3 h, “6” = 3-4 h, “7” = 4-5 h, “8” = more than 5 h), frequency of checking the phone per day, and likeliness of phone use while being with the child.

#### Set of Toys Free Play

**Figure 1:**
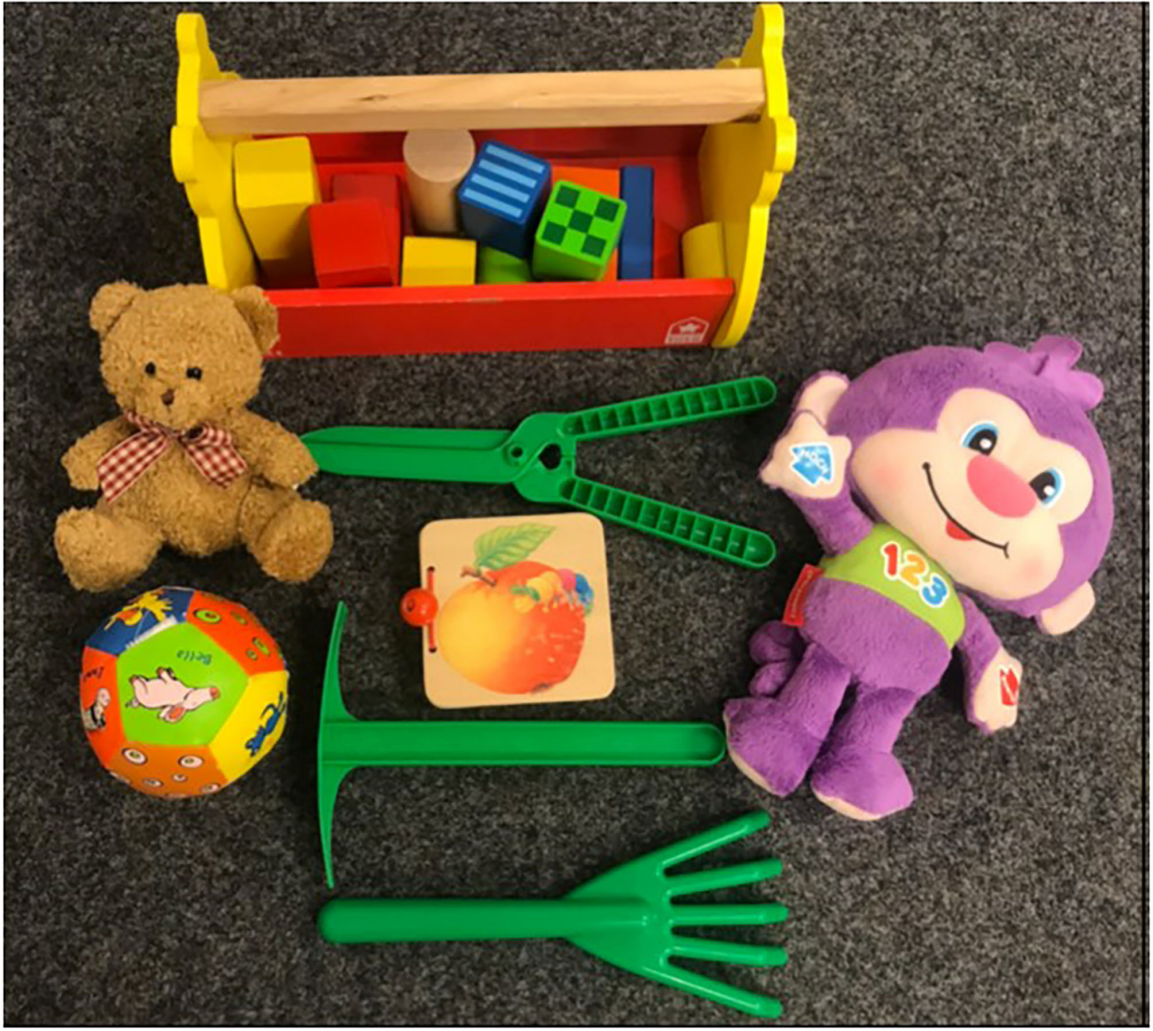
Set of toys used during free play.

Mother-child dyads received a standardized set of toys for the free play consisting of building blocks, a ball, two soft toys, a wooden book and three gardening toys (see Figure 1).

#### Smartphone

A Samsung Galaxy J5 DUOS^®^ connected to the internet was used by parents to answer the questions during interruption periods. We only put the SMS-icon on the front page so that operation was as easy as possible. The tone for incoming messages was set to “Charming Bell.”

#### Paper-Pencil

In the paper-pencil condition, mothers received the questionnaire on paper on a clipboard that mothers filled out using a pencil when the experimenter sounded a bell.

### Procedure

Mothers completed the CAFE media questionnaire online before coming into the lab for the experiment. Upon arrival, there was a brief warm-up so the child could get used to the surroundings. The experimenter explained the experiment using a cover-story and the procedure to the mother and obtained informed consent. All mothers were unaware of the reasons for the interruption and hypotheses of the study. The room was divided into a testing room and a control room by a curtain where the experimenter controlled the cameras. There were three cameras filming the room from different angles.

After a warm-up phase with the experimenter, mothers received a book that they read with their child for 5 min. Afterwards, there was a 5 min break where the experimenter entered the room again and interacted with mother and child. Then, mother and child participated in a demonstration phase where the mother demonstrated novel actions to her child. These results are not reported here. After another 5 min break the experiment started and mothers were instructed to play with their child, as they would normally do at home for 3 min. Mother and child sat on a play mat on the floor and received a box of toys (Figure 1). In the smartphone condition, mothers also received a smartphone and in the paper-pencil condition a chart with the questionnaire and a pencil. “While you are playing, I will use an SMS [smartphone group]/a bell [paper-pencil group] to advise you to carefully answer the questions about your situation. Then please just keep playing.” The experimenter then went behind the curtain and the dyads played together for 3 min until the experimenter interrupted the play. For the smartphone condition the experimenter sent a text and for the paper-and-pencil condition the experimenter rang a bell.

Mothers in the smartphone condition received a text and responded to questions on the phone during the 2 min interruption period. The questionnaire was active for 2 min and would close after this time in order to control the amount of time that mothers were given to fill out the questions. Mothers in the paper-pencil conditions responded to the same questions on paper when the experimenter rang a bell. The experimenter indicated to resume playing by ringing the bell again. Afterwards, mothers and children had another 3 min free play. Toys were present during the interruptions and children could move freely around the room like in Myruski et al. (2018) but unlike (Konrad et al., 2021). In the control condition, there was no interruption and mothers and children had a free play episode for 8 min to control for natural changes in maternal behavior over time. At the end of the session, mothers were debriefed about the nature of the study.

### Video Coding

#### Maternal Behavior

Maternal behavior was coded offline from videos for each phase by three of the authors (phase 1 free play, phase 2 interruption, phase 3 free play 2). The coding scheme for the maternal interaction quality during free play was based on existing coding schemes and consisted of 16 variables (see Table 1; Dixon et al., 1984; Fiese, 1990; Wagner et al., 2017). In accordance with the Play-PAB coding scheme (Wagner et al., 2017), the variables were coded on a rating scale from 0 to 4, with the verbal anchoring of the scales mostly varying from 0 (never) to 4 (frequently or constantly). The variables “initiate interaction” and “direct attention” were coded as absolute frequencies and for the statistical analysis, they were calculated as frequencies per minute since the three phases were of different lengths. We aimed to partially replicate existing scales so we used the original scales and then conducted a factor analysis on our data to test whether factors emerged using the combined scales. Intraclass correlations (ICCs) were used as a measure of inter-rater reliability and are displayed in Table 1. The following benchmarks were applied: > 0.9: Excellent, > 0.8: Good, > 0.7: Acceptable, > 0.6: Questionable, > 0.5: Poor, and < 0.5: Unacceptable (George and Mallery, 2003). Data were coded by a primary coder and a second independent rater who as a trained masters student blind to the hypotheses. Our initial apriori reliability check (the inter-rater reliability, 16.7% of the videos, *n* = 9 of 54) revealed that the apriori reliability was excellent to acceptable except for dynamic affect, ICC = 0.06, and criticism, ICC = 0.42). A review of the literature (Hallgreen, 2012) indicated that reliability can be lower than expected due to low base rates and that then doing an additional reliability check might increase reliability of those codes. We decided to code 5 additional videos for reliability (final reliability based on 25.9% of the data (*n* = 14 of 54) and reliability remained high for the codes overall but increased to acceptable levels for dynamic affect, ICC = 0.73, criticism, ICC = 0.76). We therefore included dynamic affect and criticism in a second factor analysis. Although the pattern of results did not change after adding the additional codes, we included them in order to include as many aspects of parenting as possible. Inclusion of these two codes also allows our data to be more consistent with prior literature examining parent-child interactions in similar studies.

**Table 1:** Coding scheme for maternal behavior.

**Code**	**Description**	**ICC**
Instruct	0 = The mother never gives instructions to the child 1 = 1 to 2 instructions 2 = 3 to 4 instructions 3 = 5 instructions 4 = The mother gives frequent or constant instructions	0.89
Direct attention	Mother directs the child's attention. For example, drawing attention to an object by pointing at it, labeling the object, instructing the child to get an object, or moving the child's hand toward an object	0.81
Initiate interaction	The mother initiates the interaction or makes a social offer to the child.	0.73
Responsiveness	0 = The mother never or rarely behaves responsively 1 = The mother is occasionally responsive but for less than half the time 2 = The mother is responsive about half the time 3 = The mother is often responsive, but not always 4 = The mother behaves responsively frequently or consistently	0.89
Dynamic emotional response	0 = The mother never or rarely shows an excited or energetic mood 1 = The mother occasionally shows an excited or energetic mood, but less than half the time 2 = The mother shows an excited or energetic mood for about half the time 3 = The mother often shows an excited or energetic mood, but not always 4 = The mother frequently or constantly shows an excited or energetic mood	0.73
Reciprocity	0 = Mother and child are never or rarely involved in joint activities 1 = Mother and child are occasionally involved in joint activities 2 = Mother and child are involved in joint activities for approximately half of the time 3 = Mother and child are often involved in joint activities 4 = Mother and child are frequently or consistently involved in joint activities	0.89
Praise	0 = The mother never praises the child 1 = 1 to 2 occasions with implied praise; the praise can be weak or inauthentic 2 = 3 to 4 occasions of indirect praise and/or 1 to 2 occasions with clear praise 3 = 5 occasions with indirect praise and/or 3 to 4 occasions with clear praise 4 = The mother praises the child frequently or constantly	0.90
Criticism	0 = The mother never criticizes the child 1 = 1 occasion with slight rejection or criticism 2 = 2 occasions with slight rejection or criticism and/or 1 occasion with clear rejection or criticism 3 = 3 occasions with slight rejection or criticism and/or 2 occasions with clear rejection or criticism 4 = The mother shows frequent or constant rejection or criticism	0.75
Verbalize	0 = The mother never makes neutral comments about the child's activity and mood 1 = The mother makes 1 to 2 neutral comments about the child's activity and moo 2 = The mother makes 3 to 4 neutral comments about the child's activity and mood 3 = The mother makes neutral comments about 5 statements the child's activity and mood 4 = The mother often or consistently makes neutral comments about the child's activity and mood	0.89
Rebukes	0 = the mother /expresses to the child no corrective/admonishing behavior 1 = 1 occasion with slight corrective/admonishing behavior 2 = 2 occasions with slight and/or 1 occasion with distinct corrective/admonishing behavior 3 = 3 occasions with slight and/or 2 occasions with clear corrective/admonishing behavior 4 = The mother expresses frequent or constant corrective /admonishing behavior	0.92
Interference	0 = The mother never interferes with the ongoing activity of the child 1 = 1 occasion with slight interference 2 = 2 occasions with slight interference and/or 1 occasion with clear interference 3 = 3 occasions with slight interference and/or 2 occasions with clear interference 4 = The mother interferes frequently or consistently in the ongoing activities of the child	0.96
Verbal threat	0 = The mother never utters verbal threats 1 = 1 occasion with mild verbal threat 2 = 2 occasions with mild and/or 1 occasion with clearer verbal threat 3 = 3 occasions with mild and/or 2 occasions with clear verbal threat 4 = The mother utters frequent or constant verbal threats	1.0
Anger	0 = The mother never shows anger or hostility toward the child 1 = 1 occasion of slight anger or hostility 2 = 2 occasions with slight anger or hostility and/or 1 occasion with moderate anger or hostility 3 = occasions with slight and/or 2 occasions with moderate anger or hostility; the mother generally shows slight anger or hostility toward the child 4 = The mother shows frequent or constant anger or hostility the child	0.97
Flat affect	0 = The mother never shows shallow affect/emotional withdrawal 1 = The mother occasionally shows shallow affect/emotional withdrawal, but for less than half the time 2 = The mother shows shallow affect/emotional withdrawal, for about half the time 3 = The mother often shows shallow affect/emotional withdrawal, but not always 4 = The mother shows frequent or persistent shallow affect/emotional withdrawal	0.46
Impatience	0 = The mother never shows impatience with the child 1 = 1 occasion with a slight impatience 2 = 2 occasions with slight impatience and/or 1 occasion with clear impatience 3 = 3 occasions with slight impatience and/or 2 occasions with clear impatience 4 = The mother shows frequent or constant impatience	0.89
Excessive control	0 = The mother never shows excessive control over task/activity 1 = 1 occasion with slightly excessive control over the task/activity 2 = 2 occasions with slightly excessive control and/or 1 occasion with excessive control over the task/activity 3 = 3 occasions of slightly excessive control and/or 2 occasions with excessive control over the task/activity 4 = The mother shows frequently or consistently excessive control over the task/activity	0.93

Scores on the variables during phase 1 were used for the factor analysis. Only variables were included in the factor analysis where at least 20% of mothers had some scores/values in any of the phases. Overall, maternal negative behavior was very low (often only visible in one or two mothers) and therefore five variables were excluded from the factor analysis (verbal threat, anger, flat affect, impatience, excessive control).

We then used several well-recognized protocols to conduct a factor analysis with the remaining 11 items. First, examination of the pattern of first order correlations showed that all items correlated at least 0.3 with at least one other item. Second, the Kaiser-Meyer-Olkin measure of sampling adequacy was 0.603, above the commonly recommended value of 0.6, and Bartlett's test of sphericity was significant [χ2_(55)_ = 177.9, *p* < 0.001]. Finally, the communalities were above 0.3 except for two variables (criticism, rebukes), further confirming that each item shared some common variance with other items. Given these overall indicators, factor analysis was deemed to be suitable with all 11 items.

A principal axes factor analysis was used. Initial eigenvalues indicated that the first two factors explained 26 and 12% of the variance, respectively. The third and fourth factors had eigen values under one, and each explained 7% of the variance. Solutions for two, three, and four factors were each examined using oblimin rotations of the factor loading matrix. The two factor solution, which explained 39% of the variance, was preferred because of: (a) its previous theoretical support; (b) the “leveling off” of eigenvalues on the screen plot after two factors; and (c) the insufficient number of primary loadings and difficulty of interpreting the third and fourth factor. The two factors shown in Table 2 were pedagogical behavior (variables: instruct, mother directs attention, interfere with the child's actions, verbalize the child's activities) and responsiveness (variables: responsiveness, reciprocity, dynamic affect). A total of four items (praise, criticism, rebukes, initiate interaction) were eliminated because they did not contribute to a simple factor structure and failed to meet a minimum criteria of having a primary factor loading of 0.30 or above on factor 1 or 2. We then calculated a mean score for each of the factors. Note that interfere with the child's actions was reverse coded before calculating the composite score.

**Table 2:** Factor analysis on maternal behavior variables.

**Variable**	**Rotated factor loading**	
	**Pedagogical behavior**	**Responsiveness**
Criticism	0.04	−0.22
Rebukes	0.27	0.04
direct attention	**0.38**	0.20
initiate interaction	0.24	0.19
Responsiveness	−0.15	**0.61**
Interference	**0.71**	−0.11
instruct child	**0.67**	0.04
Verbalize	**−0.58**	0.13
Praise	−0.09	0.07
Reciprocity	0.03	**0.82**
Dynamic affect	0.29	**0.41**
**Eigenvalues**	**2.87**	**1.37**

*N = 54. The extraction method was principal axis factoring with an oblimin (Promax with Kaiser Normalization) rotation. Factor loadings 0.30 or above are in bold*.

We hypothesized that operating a smartphone use might be more absorbing than filling out a paper sheet, and therefore coded the absorption of maternal attention during the interruption. For this purpose, phase 2 (the 2 min interruption phase for the experimental groups) was divided into four 30-s blocks. In these four blocks, absorption with the questionnaire was assessed on a 3-point rating scale (1 = occasional attention to the questionnaire/changing attention between child and questionnaire; 2 = occasional attention to the questionnaire/monitoring the child; 3 = exclusive attention to the questionnaire/no interaction with the child; based on Abels et al., 2018). Level 3 represented the highest degree of maternal distraction. A second rater coded 15.8% (*n* = 6 of 38) of the mothers. The ICCs were excellent (ICC = 0.91). For the final analysis, the variable maternal absorption during the interruption was calculated as the mean of the four coded absorption values.

#### Child Behavior

A coding scheme by Myruski et al. (2018) was modified and expanded to assess child behavior. The coded variables of child behavior analyzed here were: positive social bids to the mother (the child tries to get the mother's attention physically or vocalically, in a positive or neutral way), negative social bids to the mother (the child tries to get the mother's attention physically or vocalically in a negative way), prohibited behavior (the child does something that the mother or the experimenter has forbidden beforehand, or something that the child knows is forbidden), negative affect (negative expression or vocalization; the child protests, withdraws herself/himself or cries), and toy engagement (child plays alone with the toys provided or with other objects that belong to the play situation, e.g., chair, box, blanket). The variables negative affect, and toy engagement were coded as duration in seconds, all other variables were coded as absolute frequencies. Times when negative affect/toy engagement were not visible on the video (e.g., face was hidden) was coded as “non-codable” in seconds and later relativized for the duration of the variable. All variables were included in the analysis since at least 20% of children had some scores/values in any of the phases.

Videos were coded by one author. A second rater who was another author coded 20.4% of the videos (*n* = 11 of 54). The following benchmarks were applied: > 0.9: Excellent, > 0.8: Good, > 0.7: Acceptable, > 0.6: Questionable, > 0.5: Poor, and < 0.5: Unacceptable (George and Mallery, 2003). The ICCs were acceptable (negative affect, 0.69) to good (positive social bids, 0.88; negative social bids, 0.85; prohibited behavior, 0.95; toy engagement, 0.90). For the final analyzes, the frequency variables were calculated as frequencies per minute and the duration variables as a percentage of the time, since the three phases were of different lengths.

### Statistical Analyses

For hypothesis 1, two mixed ANOVAs, with phase as a within-subject factor (phase 1, phase 2, phase 3) and condition as a between subject-factor (smartphone, paper-pencil, no-interruption) with responsiveness and pedagogical behavior as dependent variables were calculated. For hypothesis 2, five mixed ANOVAs, with phase as a within-subject factor (phase 1, phase 2, phase 3) and condition as a between subject-factor (smartphone, paper-pencil, no-interruption) with positive social bids per minute, negative social bids per minute, prohibited behavior per minute, negative affect and toy engagement as a percentages of the time, were calculated. Follow-up ANOVAs at each phase were conducted to disentangle interactions. We used the software IBM SPSS Statistics 25 (IBM Corp., 2017) for the analyses.

## Results

### Descriptive Statistics for Maternal Mobile Device Use

For a typical weekday, 13% of mothers reported to use their phone <30 min, 36% use it 30–60 min, 32% use it 1–2 h, and 15% use it 2–3 h, 2% 3–4 h, and 2% 4–5 h (*n* = 47 reporting). This indicates that on average mothers used their smartphones for between 30 min and 2 h per day, consistent with other reports of daily maternal smartphone usage (e.g., Yuan et al., 2019; Barr et al., 2020). 8.5% of mothers reported that they typically check their smartphone every 3 h, 30% every 2 h, 34% every hour, 19% every half an hour, 8.5% every 15 min (*n* = 47 reporting). Seventeen percent of mothers indicated that they never use the smartphone during play with the child, 32% indicated that it is not very likely, 26% indicated neutral, 21% indicated that it is likely, and 4% indicated that it is very likely (*n* = 47 reporting).

There was no difference between conditions in how much mothers reported using their phone during a typical weekday, *F*_(2, 45)_ = 0.2, *p* = 0.80, ηp2 = 0.01. Furthermore, mothers from the three groups did not differ in how likely they would use their smartphone in front of their child, *F*_(2, 45)_ = 0.7, *p* = 0.49, ηp2 = 0.03, or how often they check their smartphone per day, *F*_(2, 45)_ = 1.4, *p* = 0.27, ηp2 = 0.06.

### Absorption During the Interruption

Mothers were occasionally absorbed by the questionnaire, both in the smartphone- (*M* = 2.06, *SD* = 0.52, *n* = 18) as well as the paper-pencil group (*M* = 2.2, *SD* = 0.73, *n* = 20). Absorption did not differ between mothers who used a smartphone and mothers who used the paper-pencil questionnaire, *t*_(36)_ = 0.45, *p* = 0.65. We found no significant correlations between self-reported habitual maternal mobile device use and absorption in the smartphone or in the paper-pencil condition (biggest *r* = 0.26, *p* = 0.30). The more mothers were absorbed by the questionnaire, the less they exhibited responsiveness and pedagogical behavior in both the smartphone (*r* = −0.72, *p* = 0.001; *r* = −0.59, *p* = 0.01) and in the paper-pencil conditions (*r* = −0.84, *p* < 0.001; *r* = −0.80, *p* < 0.001).

### Maternal Behavior

**Figure 2:**
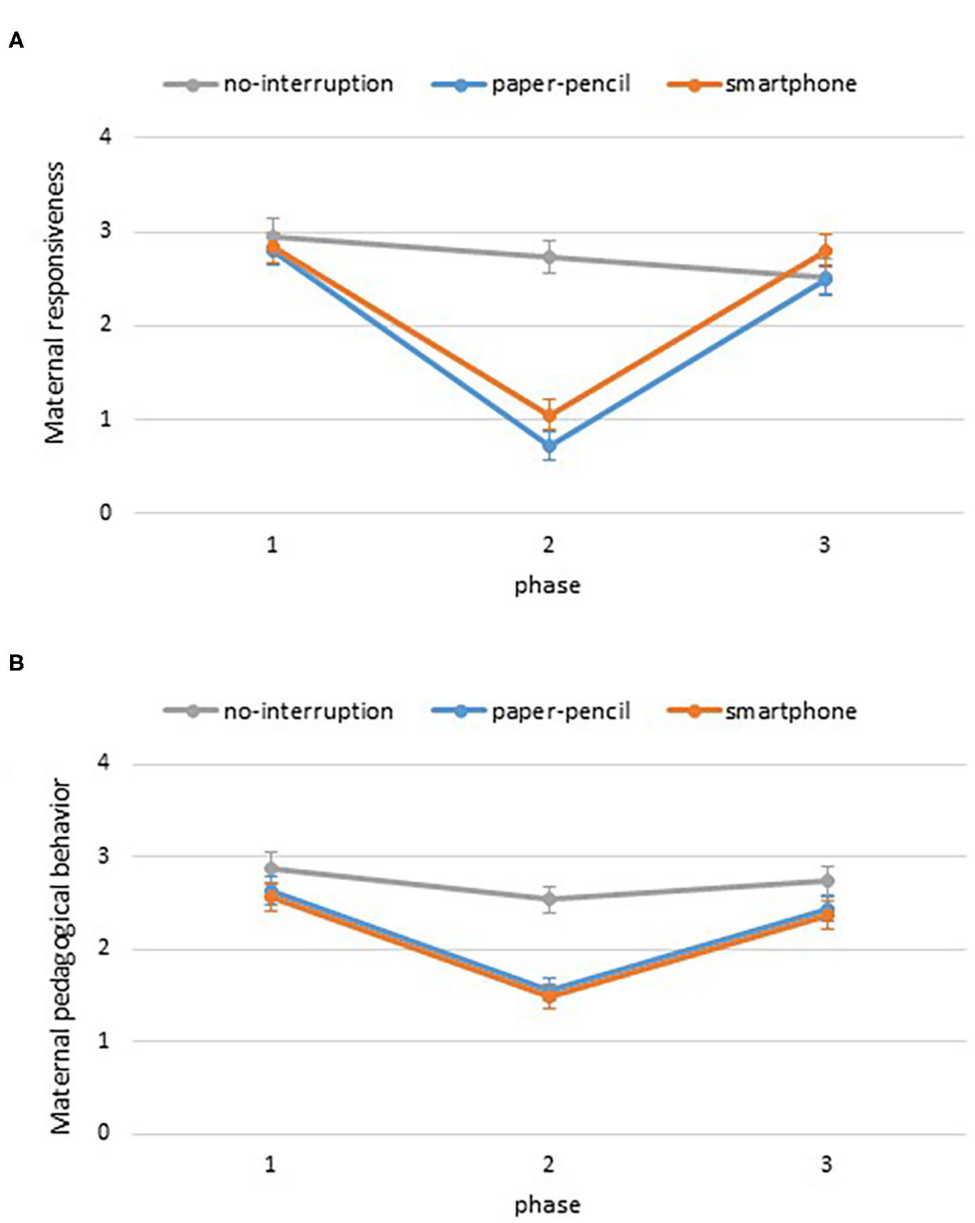
Maternal responsiveness **(A)** and pedagogical behavior **(B)** as a function of phase and condition. Error bars are SE of M. ^*^p < 0.05.

Figure 2A displays maternal responsiveness as a function of condition and phase. A mixed-ANOVA with phase (play, interruption, play) as a within-subject factor and condition (smartphone, paper-pencil, no-interruption) as a between-subject factor revealed significant main effects for phase, *F*_(2, 102)_ = 111.4, *p* < 0.001, ηp2 = 0.67, and condition, *F*_(2, 51)_ = 7.3, *p* = 0.002, ηp2 = 0.22. The main effects were qualified by a significant interaction between condition and phase on maternal responsiveness, *F*_(4, 102)_ = 26.1.4, *p* < 0.001, ηp2 = 0.51. To disentangle the interaction, we conducted follow-up one-way between-subjects ANOVAs at each phase. These analyses showed that there was no difference between conditions in maternal responsiveness before [*F*_(2, 51)_ = 0.2, *p* = 0.79, ηp2 = 0.01] and after the interruption [*F*_(2, 51)_ = 0.1, *p* = 0.38, ηp2 = 0.04]. Conditions differed during the interruption period [*F*_(2, 51)_ = 41.1, *p* < 0.001, ηp2 = 0.62] and Bonferroni *post-hoc t*-tests indicated that mothers were more responsive to their child in the no-interruption condition compared to the smartphone (*Mdiff* = 1.67, *p* < 0.001) and paper-pencil conditions (*Mdiff* = 2.01, *p* < 0.001) (Figure 2A), but responsiveness did not differ between the paper-pencil and smartphone conditions, *Mdiff* = 0.34, *p* = 0.42. To capture within-subject changes, we conducted repeated-measures ANOVAs for each condition. Maternal responsiveness decreased across phases in the no-interruption condition, *F*_(2, 30)_ = 3.9, *p* = 0.031, ηp2 = 0.21. Pairwise comparisons indicated that mothers showed marginally less responsiveness in phase 3 compared to phase 1 (*Mdiff* = 0.44, *p* = 0.051). Maternal responsiveness changed significantly in the paper-pencil condition across phases, *F*_(2, 38)_ = 82.1, *p* < 0.001, ηp2 = 0.81. Pairwise comparisons indicated that mothers showed more responsiveness in phase 1 compared to phase 2 (*Mdiff* = 2.08, *p* < 0.001), and less responsiveness in phase 2 compared to phase 3 (*Mdiff* = −1.77, *p* < 0.001). Likewise, maternal responsiveness changed significantly in the smartphone condition across phases, *F*_(2, 34)_ = 77.9, *p* < 0.001, ηp2 = 0.82. Pairwise comparisons indicated that mothers showed more responsiveness in phase 1 compared to phase 2 (*Mdiff* = 1.78, *p* < 0.001), and less responsiveness in phase 2 compared to phase 3 (*Mdiff* = −1.74, *p* < 0.001). These findings are consistent with our hypothesis that there would be a u-shaped function in responsiveness in the smartphone and paper-and-pencil conditions.

As shown in Figure 2B, pedagogical behavior decreases during phase two in the smartphone and paper-pencil condition, but not in the no-interruption condition. A mixed-ANOVA with phase (play, interruption, play) as a within-subject factor and condition (smartphone, paper-pencil, no-interruption) as a between-subject factor revealed significant main effects for phase, *F*_(2, 102)_ = 39.5, *p* < 0.001, ηp2 = 0.44, and condition, *F*_(2, 51)_ = 8.0, *p* = 0.001, ηp2 = 0.24. The main effects were qualified by a significant interaction between condition and phase on pedagogical behavior, *F*_(4, 102)_ = 3.6, *p* = 0.008, ηp2 = 0.12. To disentangle the interaction, we conducted follow-up one-way between-subjects ANOVAs at each phase. These analyses showed that there was no difference between conditions in maternal pedagogical behavior before [*F*_(2, 51)_ = 1.0, *p* = 0.38, ηp2 = 0.04] and after the interruption [*F*_(2, 51)_ = 1.7, *p* = 0.20, ηp2 = 0.06]. Conditions differed during the interruption period [*F*_(2, 51)_ = 19.3, *p* < 0.001, ηp2 = 0.43] and Bonferroni *post-hoc t*-tests indicated that mothers were more pedagogical to their child in the no-interruption condition compared to the smartphone (*Mdiff* = 1.05, *p* < 0.001) and paper-pencil conditions (*Mdiff* = 0.97, *p* < 0.001) (Figure 2B), but pedagogical behavior did not differ between the paper-pencil and smartphone conditions, *p* = 0.10. To capture within-subject changes, we conducted repeated-measures ANOVAs for each condition. Pedagogical behavior did not change across phases in the no-interruption condition, *F*_(2, 30)_ = 1.3, *p* = 0.29, ηp2 = 0.08. Pedagogical behavior changed significantly in the paper-pencil condition across phases, *F*_(2, 38)_ = 23.5, *p* < 0.001, ηp2 = 0.55. Pairwise comparisons indicated that mothers showed more pedagogical behavior in phase 1 compared to phase 2 (*Mdiff* = 1.08, *p* < 0.001), and less pedagogical behavior in phase 2 compared to phase 3 (*Mdiff* = −0.87, *p* < 0.001). Likewise, pedagogical behavior changed significantly in the smartphone condition across phases, *F*_(2, 34)_ = 42.4, *p* < 0.001, ηp2 = 0.71. Pairwise comparisons indicated that mothers showed more pedagogical behavior in phase 1 compared to phase 2 (*Mdiff* = 1.08, *p* < 0.001), and less pedagogical behavior in phase 2 compared to phase 3 (*Mdiff* = −0.89, *p* < 0.001). These findings once again indicated the predicted u-shaped function.

### Child Behavior

**Figure 3:**
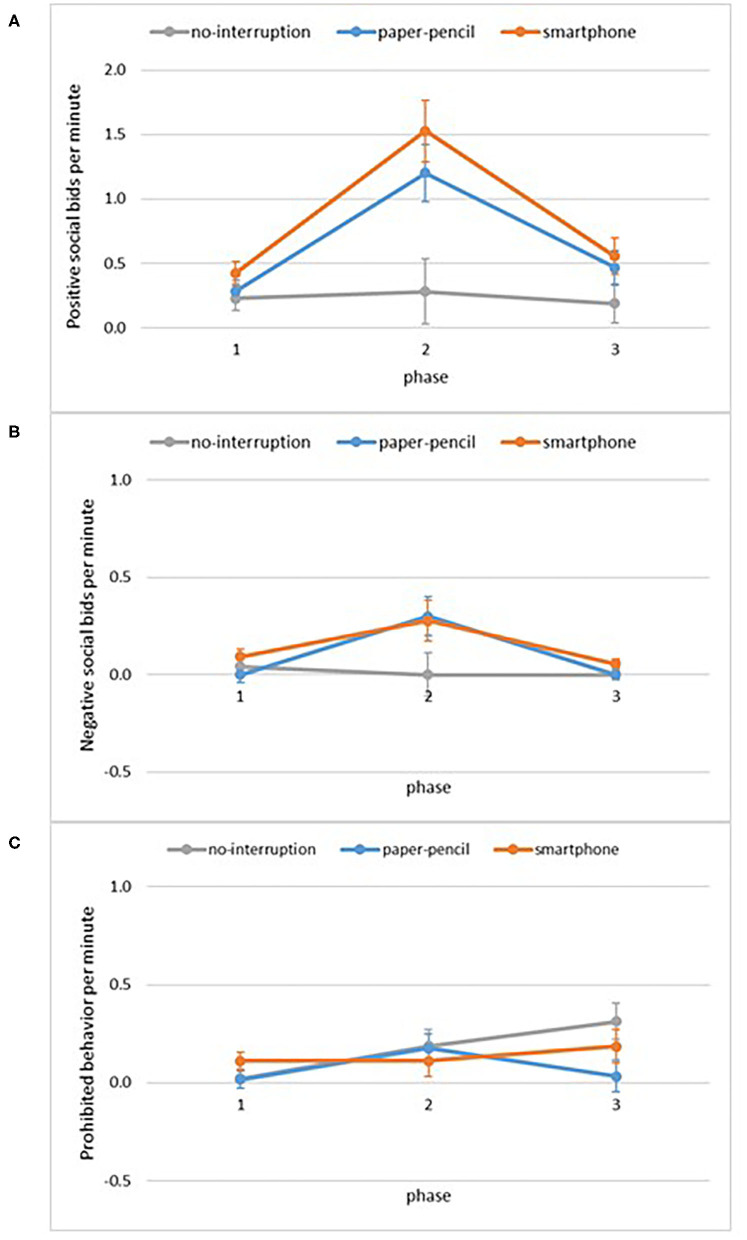
Child behaviors per minute as a function of phase and condition. **(A)** Positive social bids per minute, **(B)** negative social bids per minute, **(C)** prohibited behavior per minute. Error bars are SE of M.

Figure 3 displays child behaviors as a function of condition and phase. A mixed-ANOVA with phase (play, interruption, play) as a within-subject factor and condition (smartphone, paper-pencil, no-interruption) as a between-subject factor revealed a significant interaction between phase and condition for positive social bids, *F*_(3.12, 79.67)_ = 4.1, *p* = 0.004, ηp2 = 0.14. There were significant main effects of phase, *F*_(1.56, 79.67)_ = 20.8, *p* < 0.001, ηp2 = 0.29, and condition, *F*_(2, 51)_ = 6.2, *p* = 0.004, ηp2 = 0.20. To disentangle the interaction, we conducted follow-up one-way between-subjects ANOVAs at each phase. These analyses showed that there was no difference between conditions in positive social bids before, *F*_(2, 51)_ = 1.3, *p* = 0.28, ηp2 = 0.05, and after the interruption, *F*_(2, 51)_ = 1.7, *p* = 0.19, ηp2 = 0.06. Conditions differed during the interruption period *F*_(2, 51)_ = 6.9, *p* = 0.002, ηp2 = 0.21, and Bonferroni *post-hoc t*-tests indicated that children in the smartphone and paper-pencil conditions displayed more positive social bids toward their mother compared to the no-interruption condition (*Mdiff* = −0.92 *p* = 0.026 in paper-pencil, *Mdiff* = −1.25, *p* = 0.002 in smartphone, Figure 3A) and that these behaviors did not differ between the paper-pencil and smartphone conditions, *Mdiff* = 0.322, *p* = 0.96. That is, those in the smartphone and paper and pencil conditions attempted to re-engage the caregiver using positive bids during the interruption phase. To capture within-subject changes, we conducted repeated-measures ANOVAs for each condition. Positive social bids did not change across phases in the no-interruption condition, *F*_(2, 30)_ = 0.2, *p* = 0.81, ηp2 = 0.01. Positive social bids changed significantly in the paper-pencil condition across phases, *F*_(1.38, 26.12)_ = 10.5, *p* = 0.001, ηp2 = 0.36. Pairwise comparisons indicated that children showed more positive social bids during the interruption compared to the first free-play phase (*Mdiff* = 0.73, *p* = 0.015), and more positive social bids during the interruption compared to the second free-play phase (*Mdiff* = 0.92, *p* = 0.006). Likewise, positive social bids changed significantly in the smartphone condition across phases, *F*_(2, 34)_ = 14.9, *p* < 0.001, ηp2 = 0.47. Pairwise comparisons indicated that children showed more positive social bids during the interruption compared to the first free-play phase (*Mdiff* = 0.97, *p* = 0.003), and more positive social bids during the interruption compared to the second free-play phase (*Mdiff* = 1.10, *p* = 0.001).

A mixed-ANOVA on negative social bids with phase (play, interruption, play) as a within-subject factor and condition (smartphone, paper-pencil, no-interruption) as a between-subject factor revealed a significant main effects of phase, *F*_(1.30, 66.45)_ = 4.2, *p* = 0.011, ηp2 = 0.11, no significant main effect of condition, *F*_(2, 51)_ = 2.5, *p* = 0.089, ηp2 = 0.09, and no significant interaction effect, *F*_(2.61, 66.45)_ = 2.0, *p* = 0.16, ηp2 = 0.07. Bonferroni *post-hoc t*-tests indicated that children displayed marginally more negative social bids toward their mother during the interruption phase compared to first free play phase (*Mdiff* = −0.15, *p* = 0.06) (Figure 3B), and significantly more negative social bids during the interruption compared to the 2nd free play phase (*Mdiff* = 0.17, *p* = 0.028). The interaction effect was marginal and the pattern of results indicates that negative bids increased during the interruption phase, but did not rise to the level of statistical significance.

Prohibited behavior also changed as a function of phase, *F*_(2, 102)_ = 3.9, *p* = 0.023, ηp2 = 0.07 (Figure 3C). Bonferroni *post-hoc t*-tests indicated there were no significant differences in prohibited behavior during the interruption phase compared to the first free play phase (*Mdiff* = 0.11, *p* = 0.070), or between the 2nd free play phase compared to the interruption phase (*Mdiff* = −0.02, *p* = 1.000). There were no significant main effects of condition, *F*_(2, 51)_ = 0.9, *p* = 0.430, ηp2 = 0.3, and no interaction effect, *F*_(4, 102)_ = 2.3, *p* = 0.065, ηp2 = 0.08. That is, overall rates of prohibited behavior were low.

**Figure 4:**
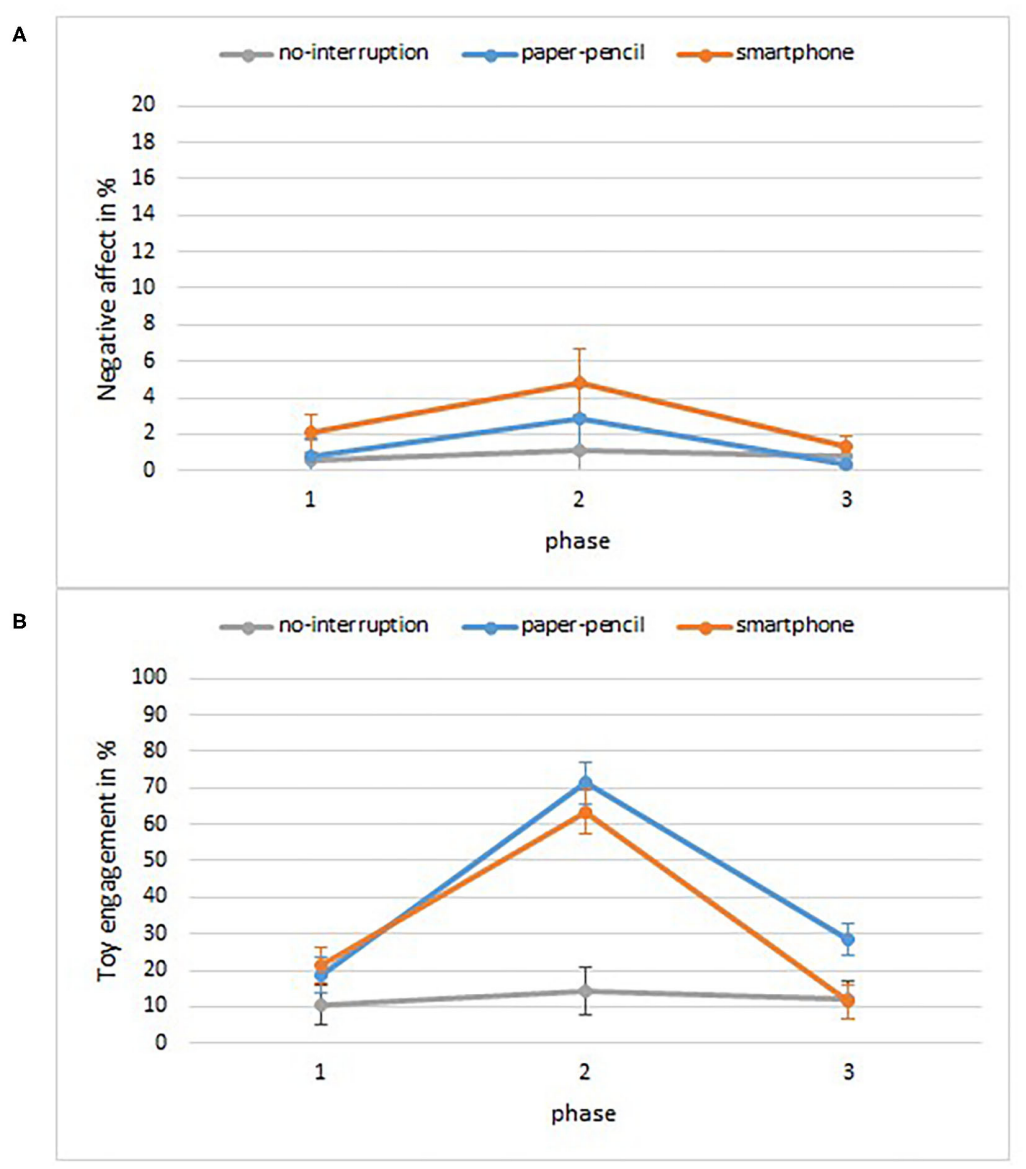
Child behaviors as percentage of the time as a function of phase and condition. **(A)** Negative affect, **(B)** toy engagement. Error bars are SE of M.

Negative affect was very low in general (see Figure 4A) but changed as a function of phase, *F*_(1.23, 62.60)_ = 4.7, *p* = 0.026, ηp2 = 0.09. Bonferroni *post-hoc t*-tests indicated that children displayed marginally more negative affect during the interruption phase compared to the first free play phase (*Mdiff* = 0.02, *p* = 0.059), but there was no significant difference between negative affect during the interruption compared to the 2nd free play phase (*Mdiff* = 0.02, *p* = 0.096). There were no significant main effects of condition, *F*_(2, 51)_ = 0.9, *p* = 0.424, ηp2 = 0.03, and no interaction effect, *F*_(2.46, 62.60)_ = 0.8, *p* = 0.49, ηp2 = 0.03. The overall low levels of negative affect make this pattern of results difficult to interpret.

A mixed-ANOVA on toy engagement with phase (play, interruption, play) as a within-subject factor and condition (smartphone, paper-pencil, no-interruption) as a between-subject factor revealed a significant main effects of phase, *F*_(2, 102)_ = 43.8, *p* < 0.001, ηp2 = 0.46, and condition, *F*_(2, 51)_ = 15.2, *p* < 0.001, ηp2 = 0.37. The main effects were qualified by a significant interaction between phase and condition for toy engagement, *F*_(4, 102)_ = 9.6, *p* < 0.001, ηp2 = 0.27. To disentangle the interaction, we conducted follow-up one-way between-subjects ANOVAs at each phase. These analyses showed that there was no difference between conditions in toy engagement before the interruption, *F*_(2, 51)_ = 1.2, *p* = 0.314, ηp2 = 0.04. Conditions differed during the interruption period *F*_(2, 51)_ = 24.6, *p* < 0.001, ηp2 = 0.49, and Bonferroni *post-hoc t*-tests indicated that children in the smartphone and paper-pencil conditions engaged with toys more compared to the no-interruption condition, *p* < 0.001 (Figure 4B) and that toy engagement did not differ between the paper-pencil and smartphone conditions, *Mdiff* = 0.08, *p* > 0.05. Furthermore, conditions differed after the interruption, *F*_(2, 51)_ = 4.3, *p* = 0.018, ηp2 = 0.15, and Bonferroni *post-hoc t*-test indicated that children in the paper-pencil engaged with the toys more than children in smartphone condition, *Mdiff* = 0.17, *p* = 0.037, and marginally more than children in the no-interruption condition, *Mdiff* = 0.17, *p* = 0.057. To capture within-subject changes, we conducted repeated-measures ANOVAs for each condition. Toy engagement did not change across phases in the no-interruption condition, *F*_(1.44, 21.62)_ = 0.2, *p* = 0.72, ηp2 = 0.02. Toy engagement changed significantly in the paper-pencil condition across phases, *F*_(2, 38)_ = 31.8, *p* < 0.001, ηp2 = 0.63. Pairwise comparisons indicated that children showed more toy engagement during the interruption compared to the first free-play phase (*Mdiff* = −0.53, *p* < 0.001), and more toy engagement during the interruption compared to the second free-play phase (*Mdiff* = 0.43, *p* < 0.001). Likewise, toy engagement changed significantly in the smartphone condition across phases, *F*_(2, 34)_ = 26.3, *p* < 0.001, ηp2 = 0.61. Pairwise comparisons indicated that children showed more toy engagement during the interruption compared to the first free-play phase (*Mdiff* = −0.42, *p* = 0.001), and more toy engagement during the interruption compared to the second free-play phase (*Mdiff* = 0.52, *p* < 0.001).

### Relations Between Maternal and Child Behaviors

We additionally examined how maternal and child behavior during each phase was related in each condition separately. There were no other significant associations between maternal behavior and child behavior than the ones reported here.

#### Smartphone Condition

The more responsive the mothers were during the first free play, the more positive social bids the children showed during the interruption (*r* = 0.58, *p* = 0.013). The more pedagogical behavior the mother showed during the second free play, the less positive social bids the child showed (*r* = −0.50, *p* = 0.034). The more responsive a mother was during the second free play, the less prohibited behavior the child showed (*r* = −0.52, *p* = 0.026).

#### Paper-Pencil Condition

The more responsive the mothers were during the first and second free play, the less toy engagement the children showed (*r* = −0.59, *p* = 0.006; *r* = −0.61, *p* = 0.004, respectively). The more pedagogical behavior the mother showed during the interruption, the less toy engagement (*r* = −0.48, *p* = 0.032) and the less negative social bids the children showed (*r* = 0.48, *p* = 0.031).

#### No-Interruption Condition

The more responsive the mothers were during the first free play, the less negative affect the children showed (*r* = −0.66, *p* = 0.005).

### Relations Between Maternal and Child Behavior to Maternal Habitual Smartphone Use

Maternal habitual smartphone use as assessed via self-report was related to maternal responsiveness and pedagogical behavior and to child behaviors in each phase.

#### Smartphone Condition

##### Maternal Behavior

The more mothers habitually check their phone per day, the less pedagogical behavior they displayed during the first free play (*r* = −0.53, *p* = 0.027, *n* = 17). Likewise, the more mothers indicated to use their smartphone during weekdays, the less pedagogical behavior they displayed during the first (*r* = −0.625, *p* = 0.007, *n* = 17) and second free play (*r* = −0.63, *p* = 0.007, *n* = 17).

##### Child Behavior

The more mothers habitually check their phone per day, the less negative affect the child displayed during the first free play phase (*r* = −0.52, *p* = 0.034, *n* = 17) and during the interruption (*r* = −0.53, *p* = 0.029, *n* = 17). The more mothers indicated to use their smartphone during weekdays, the more positive social bids the children displayed during the second free play (*r* = 0.49, *p* = 0.045, *n* = 17). The more mothers indicated to habitually use the smartphone when spending time with their child, the less toy engagement children showed during the first free play (*r* = −0.54, *p* = 0.025, *n* = 17).

#### Paper-Pencil Condition

##### Maternal Behavior

Maternal responsiveness and pedagogical behavior in any phase was not related to maternal smartphone use (biggest *r* = −0.22, *p* = 0.21, *n* = 18).

##### Child Behavior

The more mothers habitually check their phone per day, the less negative social bids the children displayed during the interruption (*r* = −0.62, *p* = 0.007, *n* = 18). Likewise, the more mothers habitually check their phone per day, the less negative affect the child displayed during the interruption (*r* = −0.65, *p* = 0.004, *n* = 18).

#### No-Interruption Condition

##### Maternal Behavior

The more mothers indicated to habitually use the smartphone when spending time with their child, the more pedagogical behavior they showed during the free play phase 1 (*r* = 0.67, *p* = 0.018, *n* = 12) and 2 (*r* = 0.86, *p* < 0.001, *n* = 12).

##### Child Behavior

Child behavior was unrelated to maternal smartphone use (biggest *r* = 0.55, *p* = 0.06, *n* = 12).

## Discussion

Our findings replicate and extend those of Myruski et al. (2018). We also found that smartphone use during a free play episode can impair maternal responsiveness and pedagogical behavior. While in the Muryski study mothers were explicitly instructed to exhibit a still face when viewing a smartphone, in the current experiment parents were instructed to respond to a text and complete a questionnaire. Our findings extend those of Myruski et al. (2018) demonstrating that the type of interruption did not matter in our study: texting was not more disruptive than writing on paper. This indicates that the decrease of interaction quality is not solely a feature of the digital media itself. That is, parents and infants responded in similar ways to a digital interruption as they did to a non-digital interruption.

Our findings are consistent with prior observational studies demonstrating that mothers were less responsive and initiated fewer activities while using smartphones than when they were not using a smartphone (e.g., Radesky et al., 2014; Hiniker et al., 2015; Radesky J. et al., 2015; Abels et al., 2018; Lemish et al., 2019; Vanden Abeele et al., 2020; Wolfers et al., 2020). The present study added to this growing body of literature demonstrating that there was no difference in maternal responsiveness prior to the interruption across experimental conditions demonstrating that it was the interruption *per se* that was interfering with the quality of the interactions. Parents exhibited very few negative behaviors during the play period and infants also demonstrated relatively low levels of negative affect which did not differ as a function of experimental condition. This is in contrast to other findings where there were reports of significant increases in negative affect and boundary testing in children during parental smartphone use (Radesky et al., 2014; Myruski et al., 2018). In the present study, the lack of negative affect may be due to the fact that all parents reported that they owned smartphones and 25% of parents reported that such interruptions were typical daily events for their infants. In addition, infants were in a novel playroom and were allowed to move around freely during the interruption period while many of the infants Myruski and colleagues studied were prelocomotive (see also Kildare and Middlemiss, 2017; Myruski et al., 2018, for similar arguments). Finally, unlike Myruski et al. (2018) study where mothers were instructed to maintain a still face and mothers in the present study were simply asked to complete a questionnaire but were not told whether they could interact with the child or not. We found that most mothers monitored their infants, looking up periodically from the phone and checking in with the infants during the interruption but spontaneously exhibited periods of still face during the interruption. Furthermore, infants played with the novel toys more during the interruption and increased their positive bids for attention whereas in the Myruski study toy engagement decreased and negative bids for attention increased. The level of absorption was consistent across paper-and-pencil and smartphone conditions but in contrast to the Myruski findings, most parents periodically checked in with their infants during the interruption. Vanden Abeele et al. (2020) also suggested that many parents may have figured out how to balance their attention between responding to the infant and to the phone. After the interruption, maternal quality rapidly returned to pre-interruption levels suggesting that these short-term interruptions may not have a lasting negative impact on maternal interactional quality, at least in the context of otherwise positive parent-child interactions.

Our findings differ from observational studies which have reported that parental responsiveness was less impaired when parents were engaged in non-digital activities such as reading a newspaper at the playground (Hiniker et al., 2015; Lemish et al., 2019). However, our results may diverge because we also experimentally controlled the activity (completing a questionnaire) between the pencil-and-paper and the smartphone groups and found that mothers were equally absorbed in completing the questionnaire regardless of whether they completed it on paper or on the smartphone. Furthermore, the level of absorption in the task on average was moderate, meaning that most mothers periodically monitored what their child was doing during the interruption (see Vanden Abeele et al., 2020 for a similar finding). That is, one of the mechanisms by which smartphones may disrupt is simply by interfering with interactions or diverting attention. The smartphone and paper-pencil interruptions were able to distract the parent from the ongoing interaction. It is likely that other everyday activities disrupt the flow of parent-child interactions, e.g., writing a shopping list. That is, prior to the introduction of smartphones there were likely activities that interrupted the daily flow of interactions and importantly what we see here is that when the interruption is short then there is a rapid return to engaged interactions. The pervasive nature of smartphone notifications is likely to be more disruptive. It is possible that when mothers interact with personal media content that is more meaningful to them and when they are not being observed in a laboratory setting that they would be more absorbed and less responsive than if they were engaged in non-digital activities (see Vanden Abeele et al., 2020). Passive sensing technology has been able to determine when parents are using smartphones and researchers are beginning to map smartphone use to children's daily activities (e.g., Barr et al., 2020). Future research should vary the type of smartphone activity to examine whether media content contributes to interactional quality but also consider non-digital interruptions to play.

These findings demonstrate that infants attempt to reconnect with their mothers during free play whenever maternal attention is diverted. During the interruption, infants played with the toys more by themselves and also increased their positive bids for attention in an attempt to re-engage their mothers. This study demonstrated that it is difficult for mothers to multi-task and during interruptions when attention is absorbed by other tasks, maternal responsiveness decreases significantly. During phase 3, the 2nd free play period mothers and infants recovered quickly after a brief interruption. The findings are consistent with a large body of research on the still face effect where vocalizations also increase during the still face phase and although we did not see significant evidence of spillover as has been reported in other studies of the still face protocol (Goldstein et al., 2009), recovery occurs during the reunion phase.

There are some limitations to the current study. We randomly assigned participants to two interruption conditions (paper-and pencil or text) but provided no explicit instructions to engage in a still face. It is therefore possible that our study may simply be comparing parents' ability to divide their attention between the questionnaire and caregiving. By using this design, we may have underestimated the unique disruptive effects of smartphones that can grab, absorb and direct attention more than other forms of interaction. Another limitation of the present study and other experimental studies is a social desirability effect. That is, parents may have been more likely to respond during the texting than in an everyday setting. For example, Vanden Abeele et al. (2020) found that smartphone use changed as a function of the observer effect; disruption from smartphone use was less when parents were consented for an observational study than when they were observed in a public setting. We also corrected our reliability estimates. Finally, although the study was powered to detect medium effect sizes, the sample was small and relatively homogenous meaning we may have missed group differences and the generalizability of these findings may be limited to well-educated samples. Future research should examine larger, more diverse samples and examine how the addition of different forms of content, the salience of the content, and the frequency of notifications, changes the absorption by parents and the impact of the smartphone interruption.

The question remains as to whether there are cohort effects for infants born during the digital age. It is quite possible that interruptions due to technoference occur at a higher rate than non-digital interruptions that occurred prior to the widespread usage of mobile devices. There are reasons to consider that this might be the case. On average parents pick up their mobile devices 67 times per day (Yuan et al., 2019). Even allowing for the fact that some of this mobile media use occurs while the baby is asleep, recent research using passive sensing and examining parent reported use of mobile devices during child routines indicates that interruptions are likely to occur frequently throughout the day (Sundqvist et al., 2009; Yuan et al., 2019; Barr et al., 2020; Radesky et al., 2020). It is not yet known, however, if parental responsiveness and child reactions to interruptions will change as a function of frequency of smartphone usage. Over time, if the parent is less responsive, the infant may be less likely to attempt to re-engage the parent. Our correlational results suggest that this could be the case. It is also possible that infants will persist in their attempts to re-engage or learn to do so only when parents are not using smartphones. That is, they may learn that use of the smartphone is a cue that their mothers are unavailable and they will learn to wait to re-engage only after the interruption. The consequences of these changes to the child's proximal environment are not known but it is feasible that language development may be disrupted by frequent, intermittent parental smartphone usage. Instrumental differences in smartphone usage may be associated with outcomes. Sundqvist et al. (2021) demonstrated that increased reports of likelihood of smartphone usage during child routines were associated with poorer language in 2-year-olds. Individual differences might predict more smartphone usage. For example, Wolfers et al. (2020) found the mothers with lower maternal sensitivity were more likely to use smartphones for longer durations of time. The directionality of this finding is unclear. Research in this area will need to consider the bidirectional communication patterns between parents and infants in order to understand the impact of technology on language development and other developmental outcomes.

Some children may be differentially susceptible to interruptions due to technoference (Piotrowski and Valkenburg, 2015). Some studies have shown that children with more difficult temperaments may be more likely to be given mobile devices by their parents as a strategy to calm them down (Radesky et al., 2016b). It is possible that infants with more difficult temperaments who find it more difficult to self-regulate may also be more susceptible to negative consequences of frequent parental use of devices and interruptions to ongoing parent-child interactions. This is an empirical question that warrants further investigation. Specifically, future research should longitudinally investigate how different patterns of technoference within families across time interacts with individual infant differences, parenting quality and child responsiveness.

In conclusion, the present study adds to a small but growing body of literature showing the mobile device usage can disrupt typical parent-child interactions. The present study also demonstrates that the pattern of results was the same whether the interruption was digital or not, at least when the same type of activity is engaged in on each medium. Although future research is warranted, these findings suggest that technoference might operate in similar ways to other types of interruptions when the same type of activity is engaged in across each type of interaction.

## Data Availability Statement

The data supporting the conclusions of this article will be made available by the authors, without undue reservation.

## Ethics Statement

The studies involving human participants were reviewed and approved by Local ethics committee, Faculty of Psychology, Ruhr University Bochum. Written informed consent to participate in this study was provided by the participants' legal guardian/next of kin.

## Author Contributions

CK conceived the study idea. CK, RB, MH, JR, and LNi contributed conception and design of the study. MH, JR, LNi, and LNe collected data. CK performed the statistical analysis. CK, MH, and RB wrote the manuscript. All authors read and approved the submitted version.

## Conflict of Interest

The authors declare that the research was conducted in the absence of any commercial or financial relationships that could be construed as a potential conflict of interest.
